# Study on improving yield and antioxidant enzyme activities in millet by rationing molybdenum and nitrogen

**DOI:** 10.3389/fpls.2024.1524347

**Published:** 2025-01-22

**Authors:** Yuan Zhao, Guoliang Wang, Min Liu, HongTao Xue, PeiYue Zhao, BaiShu Han, HuiPing Fan, Rui Wang, LiXia Wang, ErHu Guo, AiYing Zhang

**Affiliations:** ^1^ College of Agriculture, Shanxi Agricultural University, Jing Zhong, China; ^2^ Shanxi Agricultural University, Millet Research Institute, Chang Zhi, China; ^3^ Shanxi Agricultural University, The Industrial Crop Institute, Tai Yuan, China

**Keywords:** molybdenum fertilizer, nitrogen fertilizer, millet, yield, antioxidant oxidase

## Abstract

The application of appropriate nitrogen and molybdenum fertilizer can improve the growth and development of plants, increase photosynthetic efficiency, regulate active oxygen metabolism *in vivo*, maintain the oxidation balance required for normal cell growth, enhance the activity of crop antioxidant enzymes and dry matter accumulation, so as to increase crop yield. In order to investigate the effect mechanism of nitrogen fertilizer combined with foliar molybdenum fertilizer on millet yield and antioxidant enzyme activity, two nitrogen application gradients (N_0_ (0 kg/hm2) and N_1_ (75 kg/hm2) were set with millet variety Changnong 47 as material. Leaf molybdenum fertilizer Mo_0_ (0 %), Mo_1_ (0.1 %), Mo_2_ (0.2 %), Mo_3_ (0.3 %) and Mo_4_ (0.4 %) were sprayed at the joining stage. Photosynthetic parameters, chlorophyll content, antioxidant enzyme activity, dry matter accumulation and yield at the complete ripening stage were measured. After the analysis of significant difference, the results showed that the combined application of molybdenum nitrogen significantly increased the yield of millet, and the maximum yield under the Mo3 treatment was 5869.04 kg/hm_2_ under the N1 condition, which was 13% higher than that under the no fertilization treatment. The total dry matter accumulation was 36.96 g/ plant, which was 31% higher than that without fertilization. The net photosynthetic rate (Pn) and stomatal conductivity (gs) increased first and then decreased with the increase of molybdenum fertilizer concentration gradient, and reached the maximum values under N_1_Mo_3_ condition, which were 24.77 μmol•m_-2_•s_-1_ and 391.33 mol•m_-2_•s_-1_, respectively. Application of molybdenum fertilizer can improve the activities of superoxide dismutase (SOD), peroxidase (POD) and catalase (CAT) in the test samples. In conclusion, under N_1_ condition, Mo_3_ (0.3%) treatment can effectively improve millet yield, photosynthetic characteristics and antioxidant enzyme activity. The results of this study provided theoretical basis and data support for the application of nitrogen and molybdenum fertilizer in millet production.

## 1 Introduction

Foxtail Millet (*Setaria italica*) originated in the northern regions of our country and has a long history of cultivation (Xianmin, 2022).Millet is a C4 plant in the genus Setaria of the grass family. It was domesticated and cultivated about 8,000 years ago. In the late Neolithic Age, millet gradually replaced glutinous broom corn and became an important food in northern China (Shunguo et al., 2022). According to the Food and Agriculture Organization of the United Nations (FAO), the global production of millet accounts for about 1-2% of all cereals, although it is relatively small in the global cereal production, but in some countries and regions, especially in India, some countries in Africa and a small part of China, it is one of the important staple foods (FAO, 2020). In traditional agricultural areas, especially in sub-Saharan Africa and India, millet is a daily staple, often joining other crops (such as rice, wheat, beans) to form a diverse diet. It is not only a basic source of nutrition, but also is often used to make wine, pastries and other foods, enhancing the diversity of the diet (Shivani et al., 2024).Millet has high nutritional value, good adaptability, drought resistance, flood resistance and barren resistance. At present, India faces both nutrition and agriculture problems. The largest area of farmland with irrigation capacity has been basically used up, while the area of dry land is constantly expanding. To achieve nutritional security and the Sustainable Development Goals (SDG), millet is a sustainable solution with high nutritional content, bioactive and medicinal properties, and climate resilience (Mazumder et al., 2024). Therefore,Millet is therefore an important choice for eco-agriculture and sustainable food sources to combat hunger and build corrosion-resistant agri-food systems in a rapidly changing global climate (Shuai et al., 2021; Vishakha et al., 2024). The increased application of nitrogen, phosphorus, and potassium fertilizers can promote the improvement of crop yields, provided that the influence of many factors is taken into account. These factors include the environment, and excessive application will lead to soil compaction, changes in the physical and chemical properties of the soil, shrinking the porosity of the soil, and other problems, which are not conducive to the development of agriculture (Xu et al., 2022).

The advancement of sustainable agriculture has led to the emergence of more sophisticated fertilization techniques, with the integrated use of nitrogen and micro-nutrient fertilization representing a significant development in this regard. Trace elements, including boron (B), iron (Fe), zinc (Zn), copper (Cu), manganese (Mn), molybdenum (Mo). And others, despite their low concentration in plant tissue, are essential for optimal plant growth and metabolic processes (Shuai et al., 2021; Xu et al., 2022). In recent years, there has been a considerable amount of research conducted on the use of nitrogen fertilizer with micro-nutrient fertilization in barley, rice, wheat, and other crops (Xiaojia et al., 2023; Meilan et al., 2023). The application of nitrogen and silicon fertilizers has been demonstrated to enhance the height of wheat plants and augment their capacity to capture light energy. Furthermore, a “dilution effect” has been observed between leaf silicon concentration and nitrogen, with a notable negative correlation between biomass and leaf silicon concentration (Bobrenko et al., 2022).*S. Dhaliwal* discovered that the application of iron, zinc, and nitrogen-manganese fertilizers can enhance the yield and quality of wheat (Minglong et al., 2023). The chlorophyll content and photosynthetic characteristics of maize were significantly increased under the conditions of nitrogen fertilizer and iron fertilizer, and the nitrogen absorption and utilization ratio and related nitrogen invertase activities of maize were significantly increased (Felix et al., 2022). The application of boron and nitrogen fertilizers at the small trumpet stage resulted in increased plant height, stem thickness, leaf area per plant, and dry matter accumulation. Additionally, these treatments mitigated the effects of shading on tip length, spike grain number, and grain weight. The degree to which these effects were mitigated varied depending on the specific treatment (Dhaliwal et al., 2014). The application of micronutrient fertilizer in accordance with traditional fertilization practices can enhance the quality of rice seedlings, which is conducive to rice transplanting. Furthermore, it can promote rice tillering, improve tillering capacity, elevate the chlorophyll content in leaves, reinforce photosynthesis, and facilitate the formation and accumulation of dry matter. This can notably augment the number of harvested spikes and the rate of fruitfulness, thereby achieving increased yields (Jamal et al., 2022). The application of nitrogen and zinc fertilizers resulted in a significant enhancement in the height, yield, and harvest index of sorghum plants (Yakun et al., 2020). Liqiang Dong et al. found that reducing nitrogen fertilizer and increasing silicon fertilizer could improve rice yield and lodging traits (Meng et al., 2021).

In recent years, molybdenum as a trace element to promote crop nitrogen absorption and transformation has gradually entered the field of vision of researchers (Kumar et al., 2023; QiangDong et al., 2024). Although the critical value of molybdenum deficiency in plants is low, the concentration of tolerance for high molybdenum is very strong, and the difference between molybdenum deficiency and molybdenum poisoning is large, the difference can be 10^4^ times (Tingting et al., 2021). The average content of molybdenum in the global soil is 2.3 mg/kg, while the average content of total molybdenum in China’s soil is 1.7 mg/kg, which is slightly lower than the global average level, and most of it is concentrated in about 2mg/kg.The provision of molybdenum to plants has been demonstrated to enhance vegetative metabolism by improving the efficiency of both enzymatic and non-enzymatic antioxidant protection systems. This has been shown to result in an improvement in the yield and quality characteristics of aromatic rice (Xiaona and Jinyun, 2019). The application of molybdenum fertilizer has been demonstrated to enhance the chlorophyll content of plant leaves and the activity of enzymes within the body’s metabolic system, including peroxidase (POD), superoxide dismutase (SOD), nitrate reductase, and glutamine synthetase. This leads to an increased accumulation of dry matter in crops, which ultimately results in the desired outcome of enhanced yields (Fei and Guangchuan, 2024). Liu Zhichen et al. studied the effects of molybdenum and nitrogen interaction on winter wheat and found that molybdenum and nitrogen combined application could improve the lodging resistance of winter wheat (Liu, 2002). The application of a molybdenum and nitrogen ratio promotes the development and formation of beans, and the top spraying of molybdenum and nitrogen fertilizers are both decisive factors in improving winter bean yields (Muhammad et al., 2021). Furthermore, the application of molybdenum and nitrogen has been demonstrated to enhance the yield and nitrogen utilization of rice, tobacco, and other crops (Chaoyang et al., 2017; ZhiCheng et al., 2021).

At present, there are few studies on the effect of molybdenum nitrogen combined application on millet yield and antioxidant enzyme activity, so the study in this respect has important practical significance.In this study, the grain variety Changnong 47 was selected as the research object. Two experimental gradients of N_0_ (0 kg/hm²) and N_1_ (75 kg/hm²) were established to apply different concentrations of foliar molybdenum fertilizer during the grain nodulation period. The experimental variables were analyzed with the objective of exploring the effects of molybdenum-nitrogen fertilization on the growth and development of millet. The objective was to provide theoretical support for the application of molybdenum-nitrogen fertilization in grain cultivation and other related areas. This was to be achieved by examining the effects of molybdenum-nitrogen fertilization on photosynthetic characteristics, antioxidant enzyme activities and yields.

## 2 Materials and methods

### 2.1 Experimental materials

The foxtail millet variety Changnong 47 was selected for this study. This variety, which has been successfully commercialized, exhibits excellent quality and palatability, and is well-suited for planting in the middle and late maturing areas of south-central Shanxi Province, Shaanxi, Liaoning, and Gansu.The experiment was initiated on May 18, 2023, with a single row measuring 5 meters in length, a row spacing of 0.4 meters, and a density of 30,000 plants per square meter. According to the policy of promoting fertilizer and pesticide reduction and efficiency improvement, Two nitrogen gradients, no nitrogen N_0_ (0 kg/hm^2^) and normal nitrogen N_1_ (75 kg/hm^2^), were used in the main area of the split plot experiment design. During the cereal pulling period, nitrogen was applied as a furrow fertilizer in the form of urea (45% N), while molybdenum was provided as ammonium molybdate (NH_4_)_6_Mo_7_O_24_ 56% Mo) in the secondary zone, with five gradients of Mo_0_ (0%). Foliar sprays of the following treatments were applied at the nodulation stage: Mo_0_ (0%), Mo_1_ (0.1%), Mo_2_ (0.2%), Mo_3_ (0.3%), and Mo_4_ (0.4%). Three replications were set up for each treatment. The harvest was carried out on October 6, 2023, using a direct cutting method for the ears, which were then dried and threshed.

### 2.2 Overview of the test site

**Figure 1:**
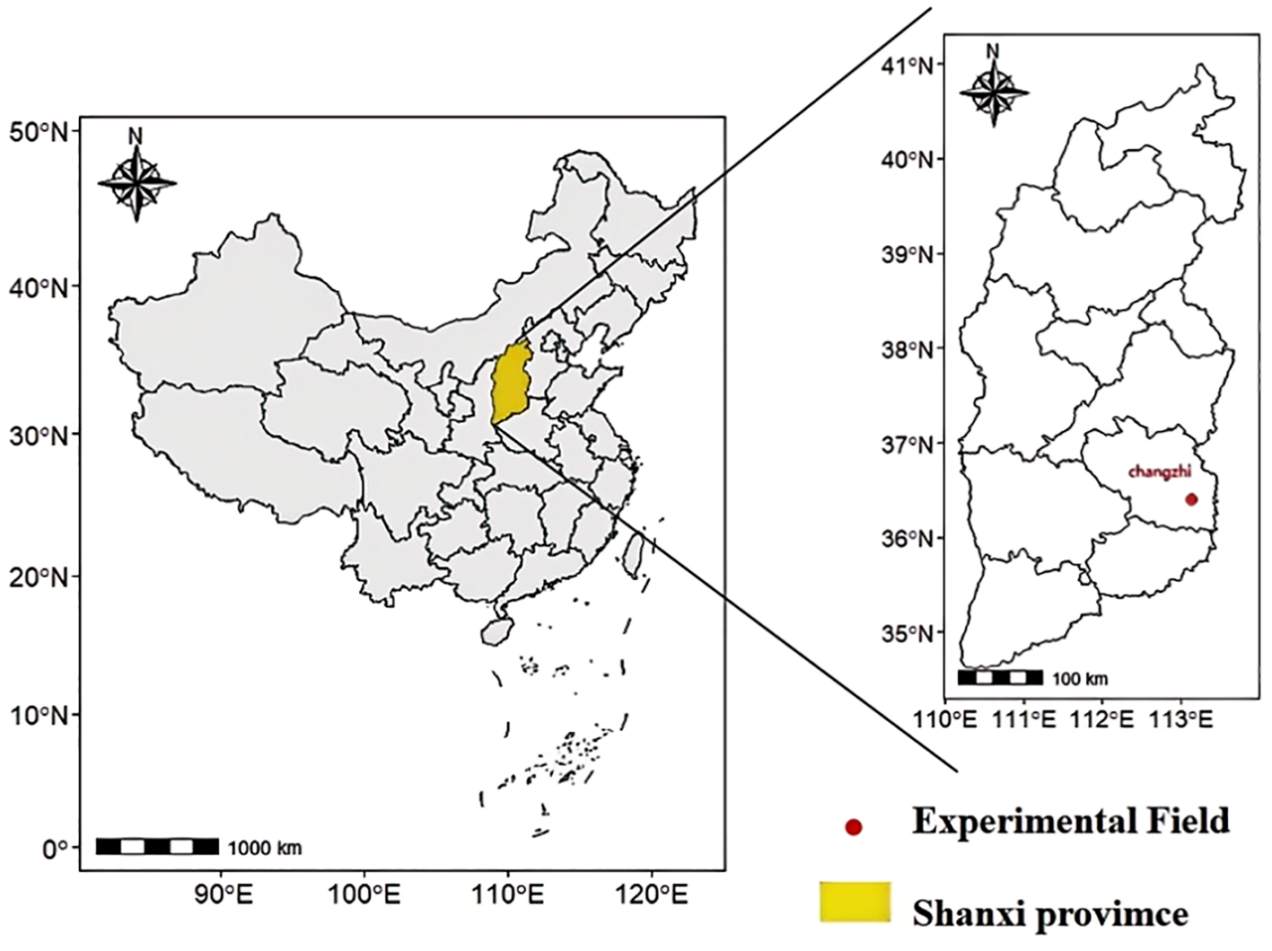
Overview of the geographic location of the test site.

The test site was situated within the experimental land of the Cereal Research Institute of Shanxi Agricultural University, Luzhou District, Changzhi City, Shanxi Province. Its geographical coordinates are longitude 113°8′27″E, latitude 36°12′57″N (**Figure 1**), and its altitude is 900 meters. The test site is located within the warm temperate zone, which is characterized by a semi-humid continental monsoon climate. The soil type at the test site was alkaline brown loam, and soil samples were obtained from the 0-20 cm soil layer prior to sowing for 10 soil element tests. The results of these tests are presented in **Table 1**, which also shows the soil element contents.

**Table 1:** Overview of the soil elements in the experiment site.

elemental	content	elemental	content
Total potassium (g/kg)	19.73	Slow-acting potassium (mg/kg)	641
Iron (mg/kg)	43705.3	Copper (mg/kg)	121
Total Nitrogen (g/kg)	1.36	Total Phosphorus (%)	0.107
Quick Potassium (mg/kg)	175	Organic Carbon (g/kg)	17.5
Manganese (mg/kg)	824	Zinc (mg/kg)	187

### 2.3 Data collection

#### 2.3.1 Agronomic traits and yield

Five cereal plants exhibiting uniform growth were selected from the middle four rows of each treatment at the grain-pulling stage seven days after fertilizer spraying, at the filling stage, and at the completion stage. The following variables were measured: plant height, spike weight, spike length, and spike thickness. The latter three variables were measured at the filling stage and at the completion stage after spiking (Filho et al., 2020; Yanhong et al., 2022). Plant height was determined with the aid of a graduated steel ruler, while spike thickness was gauged with the use of electronic vernier calipers. Yield indexes were obtained at the grain completion stage, and the completed grains were harvested in plots (5 m × 1.6 m) and then directly cut and threshed to dry. The weight of the plots was determined by electronic weighing after the removal of impurities and sieving, and the thousand grain weight was measured.

#### 2.3.2 Dry matter accumulation

Once the ripening process was complete, samples were taken from five plants of uniform growth for each treatment. The stems, sheaths, leaves, and spikes were separated, weighed individually, and then subjected to a 105°C heat treatment for 30 minutes and subsequent drying at 80°C for 72 hours. This process was employed to determine the dry matter content of each part (Zhiqing et al., 2022).

#### 2.3.3 Chlorophyll SPAD and photosynthetic activity

SPAD measurements were conducted using the SPAD-502 Plus (Konica Minolta Sensing, Inc., Japan) during the grain filling period. Photosynthesis indexes were determined by the CIRAS-2 Photosynthesis Rate Meter (PPSYSTEMS) during the filling period to ascertain the net photosynthesis rate (Pn), transpiration rate (E), and intercellular CO_2_ concentration (Ci) of the flag leaf of the grain. The net photosynthesis rate (Pn), transpiration rate (E), and intercellular CO_2_ concentration (Ci) were measured during the grain filling period.

#### 2.3.4 Antioxidant enzyme activity and malondialdehyde

Flag leaves of millet with uniform growth were sampled in the field during grout filling period, and placed in liquid nitrogen and brought back to the freezer at -80°C.

The activity of superoxide dismutase (SOD) was determined by nitro blue tetraolium (NBT) photochemical reduction method (Xiaofei et al., 2017).

Reagent:

0.05mol/L phosphate buffer (PH7.8): 21.25mL of liquid A + 228.25mL of liquid B, with a constant volume of 1L;

130mmol/L methionine (Met) solution: 1.9399g Met was filled with phosphoric acid buffer to 100ml;

750μmol/L nitrogen blue tetrazole solution: 0.06133g NBT was weighed and stored to 100mL with phosphoric acid buffer, away from light;

100μmol/L EDTA-Na_2_ solution: Weigh 0.03721g EDTA-Na_2_ and volume it with phosphoric acid buffer to 1000 ml;

20μmol/L riboflavin solution: Weigh 0.0753g riboflavin and fill it with distilled water to 1000mL, and store it away from light.

Enzyme solution preparation:remove 0.5g of leaves in a pre-cooled mortar, add 1mL of pre-cooled phosphoric acid buffer ice bath to grind into pulp, add buffer to make the final volume of 5mL. Take 1.5-2 mL and centrifuge at 1000r/min for 20min, the supernatant is the crude extract of SOD.

Color reaction: Take 5mL test tubes (requiring good transparency), 2 tubes are measuring tubes, and the other 2 tubes are monitoring, add each solution and mix.Put 1 tube monitoring in the dark, and the other tubes react for 15-20min under 4000lx sunlight (requiring the same light condition of each tube, high temperature, shorter time, and longer at low times).


(1) $$SOD=\frac{\left(A_{1}-A_{2}\right)\times V_{1}}{0.5\times A_{1}\times W\times V_{2}}$$

In Formula 1, the total activity of SOD is the absorbance of fresh heavy enzyme unit per gram (U/g), and A_1_ is the absorbance of light treatment. A_2_ is the absorbance of the sample tube; V_1_ is the total volume of sample liquid (mL); V_2_ is the sample amount (mL) at the time of measurement; W is the sample fresh weight (g).

The activity of peroxidase (POD) was determined by guaiacol method (Yuan et al., 2022).

Reagent: 50 mL phosphate buffer and 28μL guaiacol were heated and mixed on a magnetic stirrer until the guaiacol was completely dissolved. After the solution was cooled, 30%H_2_O_2_ 19 μL was added, mixed evenly and stored in a refrigerator at 4°C.

Enzyme solution preparation: Take a small number of leaves into a mortar, add 2 mL phosphate buffer (0.2 mol/L,pH 7.0) and grind into a homogenate. After all the homogenates were transferred into the 5 mL centrifuge tube, the mortar was cleaned twice with 4 mL phosphate buffer and transferred into the centrifuge tube. When centrifuged at 4°C for 20 min at 4000 r/min, the supernatant was the crude enzyme liquid.

Reaction determination: Take 3mL of reaction mixture for enzyme activity determination, add 20μL crude enzyme solution, and use the reaction mixture with 20μL phosphoric acid buffer as control. The absorbance was measured with 756 ultraviolet spectrophotometer at 470nm wavelength, and the absorbance value was recorded every 1 min, a total of 5 times. The unit μ of peroxidase activity is defined as a 0.01 change in A470 per minute.


(2) $$POD=\frac{\Delta A_{470}\times V_{t}}{w\times v_{s}\times 0.01\times t}$$

In Formula 2, △A470 is the change of absorbance during the reaction time,W is the fresh seed weight (g), V_t_ is the total volume of extracted enzyme liquid (mL),V_S_ is the volume of enzyme liquid taken during the determination (mL), and t is the reaction time (min).

malondialdehyde (MDA) content was determined by thiobarbituric acid (TBA) colorimetry (Junxia et al., 2021).

Reagents: 5% trichloroacetic acid (TAC), 0.67% thiobarbituric acid (TBA).

Preparation of enzyme solution: Take a small amount of plant tissue (leaves), mark the mass as m, add 5% TAC 5 ml, and centrifuge the homogenate obtained after grinding at 3000 r/min for 10 min.

Reaction: Use pipette gun to slowly absorb 2 ml of supernatant, not too fast, so as not to inhale the plant body, add 0.67% TBA 2 ml, mix and boil in 100°C water bath environment for 15 min, cool and then centrifuge once (10 min). The absorbance values of the supernatant at 450 nm, 532 nm and 600 nm were determined, respectively, with 2 ml distilled water plus 2 ml0.67%TBA as the control.


(3) $$\text{W}=\frac{\left[6.45\times \left(A_{532}-A_{600}\right)-0.56\times A_{450}\right]\times V}{m}$$

In the Formula 3, A450, A532 and A600 respectively represent the absorbance values at 450nm, 532nm and 600nm wavelengths, W is the content of malondialdehyde (μmol/g), V is the total volume of extract (L) (about 5×10^-3^), and m is the fresh weight of plant tissue (g).

Catalase was determined by ultraviolet spectrophotometry (Xiaobao et al., 2012).

Reagent:

Buffer solution: weigh 65.52 g Na_2_HPO_4_·12H_2_O, 2.66 g NaH_2_PO_4_·2H_2_O, 10 g polyethylpyrrolidone, dissolve in about 900 mL water, use a pH meter to check the pH, adjust to 7.80, fixed volume to 1 L; Absorb 50 mL concentrated sulfuric acid and slowly add it to about 400 mL water. After cooling, the volume is fixed to 500 mL.

Hydrogen peroxide solution:absorbed 6 mL of 30% H_2_O_2_ solution, the volume was fixed to 1 L, and the concentration was calibrated before clinical use. The oxalic acid solution was dissolved in 3.1518 g of oxalic acid (H_2_C_2_O_4_·H_2_O) and transferred to a 500 mL volumetric bottle with a concentration of 0.1 mol·L^-1^.

Reagent calibration: Calibration of potassium permanganate solution: absorb 0.1M 5 mL of oxalic acid, add 10% 5 mL of sulfuric acid, and then slowly titrate with potassium permanganate solution until purple is maintained for half a minute. Start to react slowly, each drop, must wait for its fading before dropping.

Calibration of hydrogen peroxide solution: calibrated with calibrated potassium permanganate, absorb 2.5 mL of hydrogen peroxide solution to be calibrated, add 2.5 mL of 10% sulfuric acid, and titrate with known concentration of potassium permanganate solution until the pink color remains for 30 s as the end point; Because the molar absorption coefficient of hydrogen peroxide at 240 nm wavelength is 43.6 mol·L-1·cm-1, the concentration of the solution can be calculated by measuring the absorbance of the solution at 240 nm. Absorb 2.5mL hydrogen peroxide solution to be calibrated into a 25 mL colorimetric tube, add 2.5mL 10% sulfuric acid, set the volume to 25 mL, shake well, measure the absorbance at 240 nm, and set the volume of 2.5mL 10% sulfuric acid to 25 mL as blank.

Enzyme solution preparation: The treated leaf sample was cut and mixed well, weighed with a 1% electronic balance, 2-3 g was ground, 10 mL buffer solution and a small amount of quartz sand were added, and ground for 3 min at room temperature of 25°C to form a homogenization, then transferred to a 50 mL colorimetric tube, washed and ground with a small amount of buffer solution several times, and placed in the colorimetric tube each time. Finally, the buffer solution is set to 50 mL scale. After shaking, filter or centrifuge at 4000 r·min^-1^, the clear liquid is the enzyme extract and stored in the refrigerator at 4°C for use.

Reaction: Standard curve: Take 6 50 mL colorimetric tubes, add 2 mL buffer solution and 2.5 mL sulfuric acid each, then add 0, 1, 2, 3, 4 and 5 mL hydrogen peroxide solution respectively, shake well at a constant volume and measure absorbance at 240 nm. The regression equation y=ax+b was calculated with absorbance as the independent variable x and mass of H2O2 as the dependent variable y.

Determination: Two 50 mL colorimetric tubes were taken, and 1-2 mL of enzyme extract, 2.5 mL of sulfuric acid and 2.5 mL of hydrogen peroxide were added in turn. The sample tube was successively added with 1.0-2.0 mL of enzyme extract and 2.5 mL of hydrogen peroxide, then timed immediately, kept at 25°C for 10-20 min, and immediately added with 2.5 mL of sulfuric acid to terminate the reaction. Then the charge and sample tubes were filled to 50 mL and shaken well, and the absorbance As and Ac of the charge and sample tubes were determined at 240 nm. The amount of hydrogen peroxide calculated by the regression equation of the standard curve using the difference △A between the charge and the absorbance of the sample tube is converted to the enzyme activity.


(4) $$\rho_{E}\left(mg\cdot min^{-1}\cdot g^{-1}\right)=\frac{y\times v}{v_{s}\times W\times t}$$

In Formula 4, y is △A the mass mg of H_2_O_2_ obtained through the standard curve,V is the total volume mL of the enzyme extract,Vs is the volume mL of the enzyme extract liquid absorbed during the determination,W is the weight of the plant leaf sample g, and t is the reaction time min.

### 2.4 Data analysis

Significance difference analysis was performed in IBM SPSS Statistics 27, graph rendering in Origin 2021, correlation analysis and graph rendering in RStudio.

## 3 Results and analysis

### 3.1 Agronomic characters and yield analysis

The ratio of molybdenum to nitrogen had a significant impact on grain yield, plant height, thousand kernel weight, number of grains in a spike, and kernel weight per spike (P < 0.05) (**Table 2**). The highest yield of 5869.04 kg/hm² was observed under the N_1_Mo_3_ condition, representing a significant increase of 13% compared to the no-fertilizer treatment. Under the N_1_ condition, the yields, plant heights, and thousand kernel weights demonstrated an initial increase, followed by a subsequent decline. The yields of Mo_0_, Mo_1_, Mo_2_, Mo_3_, and Mo_4_ were 5533.61, 5612.78, 5604.45, 5869.04, and 5491.94 kg/hm², the plant heights were 139.33, 137.33, 140.00, 144.33, and 138.00 cm, and the weights of 1,000 kernels were 2.97, 2.97, 3.04, 3.11, and 2.95 g, respectively.

**Table 2:** Agronomic traits and yield of grain sprayed with foliar molybdenum fertilizer at different nitrogen levels.

		height (cm)	yeild (kg/hm^2^)	thousand grain weight (g)	Number of grains in a spike	Grain weight per spike (g)
N_0_	Mo_0_	130.33 ± 0.88b	5089.84 ± 57.55c	2.85 ± 0.00g	4594.41 ± 149.32bc	13.09 ± 0.44bc
	Mo_1_	144.33 ± 3.18a	5404.78 ± 65.85b	2.88 ± 0.01f	4186.03 ± 123.27c	12.07 ± 0.30c
	Mo_2_	141.67 ± 2.73a	5181.54 ± 36.86c	2.95 ± 0.00de	4476.34 ± 62.71bc	13.22 ± 0.20bc
	Mo_3_	140.67 ± 2.40a	5333.6 ± 142.58b	3.02 ± 0.00c	4422.29 ± 99.22bc	13.36 ± 0.31b
	Mo_4_	141.67 ± 0.88a	5298.6 ± 76.16b	3.05 ± 0.01b	4318.36 ± 176.77c	13.19 ± 0.54bc
N_1_	Mo_0_	139.33 ± 2.85a	5533.61 ± 65.59b	2.97 ± 0.01d	4504.83 ± 214.12bc	13.40 ± 0.63b
	Mo_1_	137.33 ± 2.33a	5612.78 ± 38.19b	2.97 ± 0.01d	4523.07 ± 28.95bc	13.45 ± 0.08b
	Mo_2_	140.00 ± 2.52ab	5604.45 ± 34.67b	3.04 ± 0.00bc	4362.33 ± 52.38bc	13.24 ± 0.14bc
	Mo_3_	144.33 ± 0.88a	5869.04 ± 86.83a	3.11 ± 0.01a	4779.76 ± 166.00ab	14.86 ± 0.49a
	Mo_4_	138.00 ± 4.04ab	5491.94 ± 54.77b	2.95 ± 0.01e	4972.80 ± 79.59a	14.65 ± 0.24a
significant level						
N		ns	0.00^**^	0.00^**^	0.01^*^	0.00^**^
Mo		ns	0.00^**^	0.00^**^	ns	0.01^*^
N×Mo		0.034^*^	0.00^**^	0.00^**^	0.03^*^	0.03^*^

Means with different lowercase letters ± standard deviation (SD) are significantly different at the LSD test p ≤ 0.05 probability level. **p ≤ 0.001, *p < 0.05, ns not significantly different.

### 3.2 Accumulation of dry matter in all parts of the ground

Yield stability is associated with dry matter accumulation (Zhi et al., 2010). The accumulation of dry matter in the millet stem, leaf, sheath, spike, and in total was significantly greater in the treatments involving molybdenum and nitrogen in combination than in the molybdenum fertilizer alone treatment. Furthermore, the total dry matter growth exhibited a tendency to increase and then decrease with increasing molybdenum fertilizer concentration (**Table 3**). The total dry matter mass of the Mo_0_, Mo_1_, Mo_2_, Mo_3_, and Mo_4_ treatments increased by 3%, 8%, 7%, 6%, and 7%, respectively, under N_1_ condition compared with that of the N_0_ condition. Similarly, spike dry matter exhibited an increase of 4%, 7%, 12%, 4%, and 21%, respectively; leaf dry matter demonstrated an increase of 6%, 15%, 5%, 6%, and 7%, respectively; and sheath dry matter mass exhibited an increase of 6%, 14%, 6%, 12%, and 4%, respectively. The stem dry matter exhibited an increase of 1%, 7%, 1%, and 8%, respectively, in the Mo_0_, Mo_1_, Mo_2_, and Mo_3_ treatments. However, the Mo_4_ treatment demonstrated a decrease of 21%.

**Table 3:** Dry matter of each part and total dry matter mass above ground at maturitystage.

		Stem dry matter mass(g/plant)	Leaf dry matter mass(g/plant)	Sheath dry matter mass(g/plant)	Spike dry matter mass(g/plant)	Total dry matter mass (g/plant)
N_0_	Mo_0_	7.01 ± 0.03f	2.71 ± 0.02e	2.21 ± 0.12c	13.53 ± 0.04e	25.47 ± 0.47d
	Mo_1_	9.83 ± 0.07d	2.92 ± 0.01e	2.33 ± 0.22bc	15.62 ± 0.27cd	30.77 ± 0.66c
	Mo_2_	10.68 ± 0.12bc	3.33 ± 0.03bc	3.12 ± 0.17ab	14.54 ± 0.16de	31.67 ± 0.21c
	Mo_3_	10.69 ± 0.04bc	3.23 ± 0.05c	3.06 ± 0.35abc	17.82 ± 0.13ab	34.79 ± 0.62b
	Mo_4_	10.59 ± 0.03c	3.44 ± 0.06bc	2.86 ± 0.55abc	18.24 ± 0.11a	32.14 ± 0.33b
N_1_	Mo_0_	7.07 ± 0.00f	2.88 ± 0.01e	2.35 ± 0.18bc	14.08 ± 0.03e	26.39 ± 0.55d
	Mo_1_	10.56 ± 0.01c	3.42 ± 0.04bc	2.72 ± 0.04abc	16.84 ± 0.42bc	33.61 ± 0.58b
	Mo_2_	10.78 ± 0.04b	3.49 ± 0.03b	3.32 ± 0.19a	16.47 ± 0.42bc	34.08 ± 0.61b
	Mo_3_	11.57 ± 0.09a	3.45 ± 0.20b	3.48 ± 0.22a	18.47 ± 0.18a	36.96 ± 0.34a
	Mo_4_	8.75 ± 0.07e	3.72 ± 0.13a	2.98 ± 0.10abc	19.19 ± 0.14ab	34.64 ± 0.19b
significant level						
N		ns	0.00^**^	ns	0.00^**^	0.00^**^
Mo		0.00^**^	0.00^**^	0.00^*^	0.00^**^	0.00^**^
N×Mo		0.00^**^	0.00^**^	ns	0.04^*^	0.02^*^

Means with different lowercase letters ± standard deviation (SD) are significantly different at the LSD test p ≤ 0.05 probability level. **p ≤ 0.001, *p < 0.05, ns not significantly different.

### 3.3 Chlorophyll content and photosynthetic activity at filling stage

**Figure 2:**
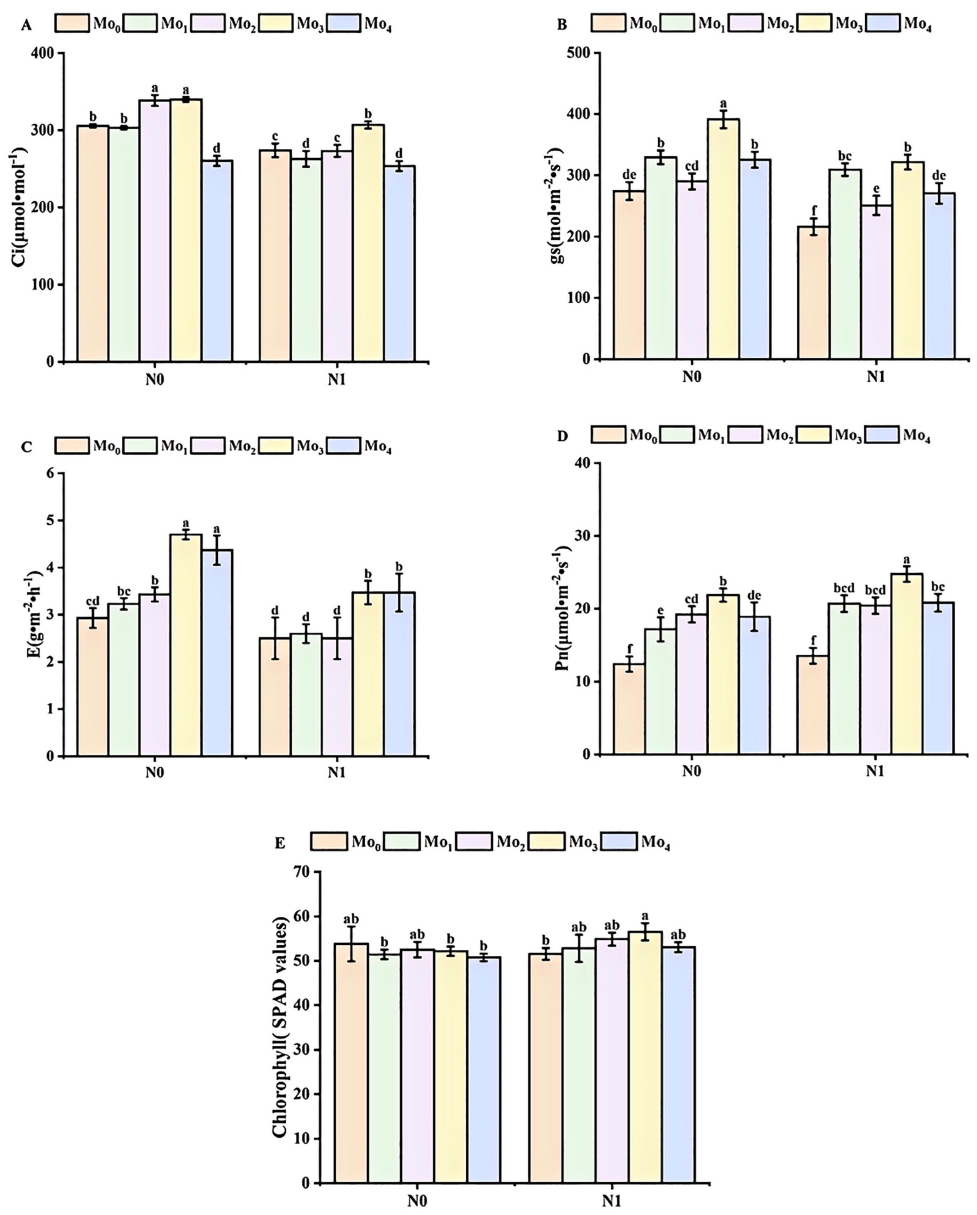
Chlorophyll content and photosynthetic activity during irrigation period. Intercellular carbon dioxide concentration **(A)**, stomatal conductance gs **(B)**; transpiration rate **(C)**, net photosynthetic rate **(D)**, and chlorophyll SPAD value **(E)**. Bar bars with different lowercase letters are significantly different by LSD test (p< 0.05).

The application of nitrogen and molybdenum fertilizers in combination led to an increase in the chlorophyll content and photosynthetic activity of cereals (**Figure 2**). In comparison to the N_0_ condition, the SPAD value increased by 3%, 4%, 8%, and 4% in the Mo_1_, Mo_2_, Mo_3_, and Mo_4_ treatments, respectively, with the exception of a 4% decrease observed in the Mo_0_ condition. Similarly, the Pn value increased by 8%, 16%, 6%, 12%, and 9% in the Mo_0_, Mo_1_, Mo_2_, Mo_3_, and Mo_4_ treatments, respectively. Additionally, the gs value decreased by 21%, 6%, 13%, 18%, and 17%, respectively. Furthermore, the application of molybdenum fertilizer resulted in a reduction in grain E, with the order of magnitude being Mo_2_ (37%) > Mo_3_ (36%) > Mo_4_ (26%) > Mo_1_ (24%) > Mo_0_ (17%).

### 3.4 Activities of POD, SOD and CAT and content of malondialdehyde in leaves at filling stage

**Figure 3:**
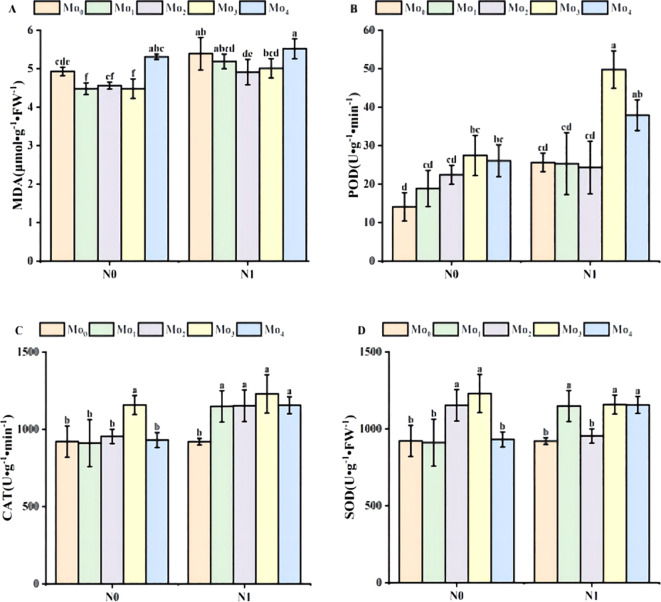
Changes in POD, SOD, CAT activities and malondialdehyde content of leaves during the period of fertilization. Malondialdehyde MDA **(A)**, peroxidase POD **(B)**; catalase CAT **(C)**, superoxide dismutase SOD **(D)**. Bars with different lower case letters are significantly different by LSD test (p< 0.05).

Under the conditions of N_1_ and N_0_, the content of malondialdehyde (MDA) under Mo_0_, Mo_1_, Mo_2_ and Mo_3_ treatments decreased first and then increased.Compared with N_0_ condition, N_1_ increased by 9%, 14%, 7% and 11% under Mo_1_-Mo_3_ treatment, respectively, and increased by 4% under Mo_4_ treatment, but the performance was not significant.This shows that the application of nitrogen fertilizer can damage the cell protective membrane lipid (**Figure 3A**). In the N_1_ condition, the content of malondialdehyde decreased at the concentration of Mo_1_-Mo_3_, indicating that molybdenum fertilizer at this concentration could enable cells to have better antioxidant capacity and cell protection mechanism, effectively reduce MDA content, and thus reduce the damage of MDA to cell membrane structure.

As the concentration of molybdenum fertilizer increased, the peroxidase (POD) exhibited a tendency to increase and then decrease (**Figure 3B**). The POD activities of the N_1_ condition were increased by 45%, 26%, 8%, 45%, and 31%, respectively, compared with that of the N_0_ condition in the Mo_0_, Mo_1_, Mo_2_, Mo_3_, and Mo_4_ treatments. This suggests that the administration of molybdenum and nitrogen can facilitate the scavenging of harmful substances, such as peroxides, within the body, thereby protecting cells from oxidative damage.

The trend of superoxide dismutase (SOD) was essentially analogous to that of peroxidase (POD) under N_0_ conditions (**Figure 3D**). Under N_0_ application, the concentrations of Mo_1_, Mo_2_, Mo_3_, and Mo_4_ decreased by 1, 2, 3, and 7%, respectively, with the exception of Mo_0_, which exhibited an 8% increase. Nevertheless, the activities of the SOD enzyme were consistently higher in the presence of molybdenum fertilizers than in the N0Mo0 control.

The data indicated a trend of increasing and then decreasing for the changes in catalase (CAT) activity. The concentration of Mo_3_ was significantly higher than that of other molybdenum fertilizers under N_1_ (**Figure 3C**). In accordance with the N_1_ conditions, the activity of CAT was observed to increase by 0%, 21%, 17%, 6%, and 19% for the Mo_0_, Mo_1_, Mo_2_, Mo_3_, and Mo_4_ treatments, respectively. The findings suggest that the cells are better able to regulate reactive oxygen metabolism within the body, thereby maintaining the oxidative balance that is essential for the normal growth and structural and functional integrity of the cells. These findings suggest that molybdenum fertilizer facilitates the cells’ regulation of reactive oxygen species metabolism, maintenance of the requisite oxidative balance for normal cell growth, reduction of hydrogen peroxide-induced oxidative damage to cells, and protection of cell structure and function.

### 3.5 Correlation analysis

**Figure 4:**
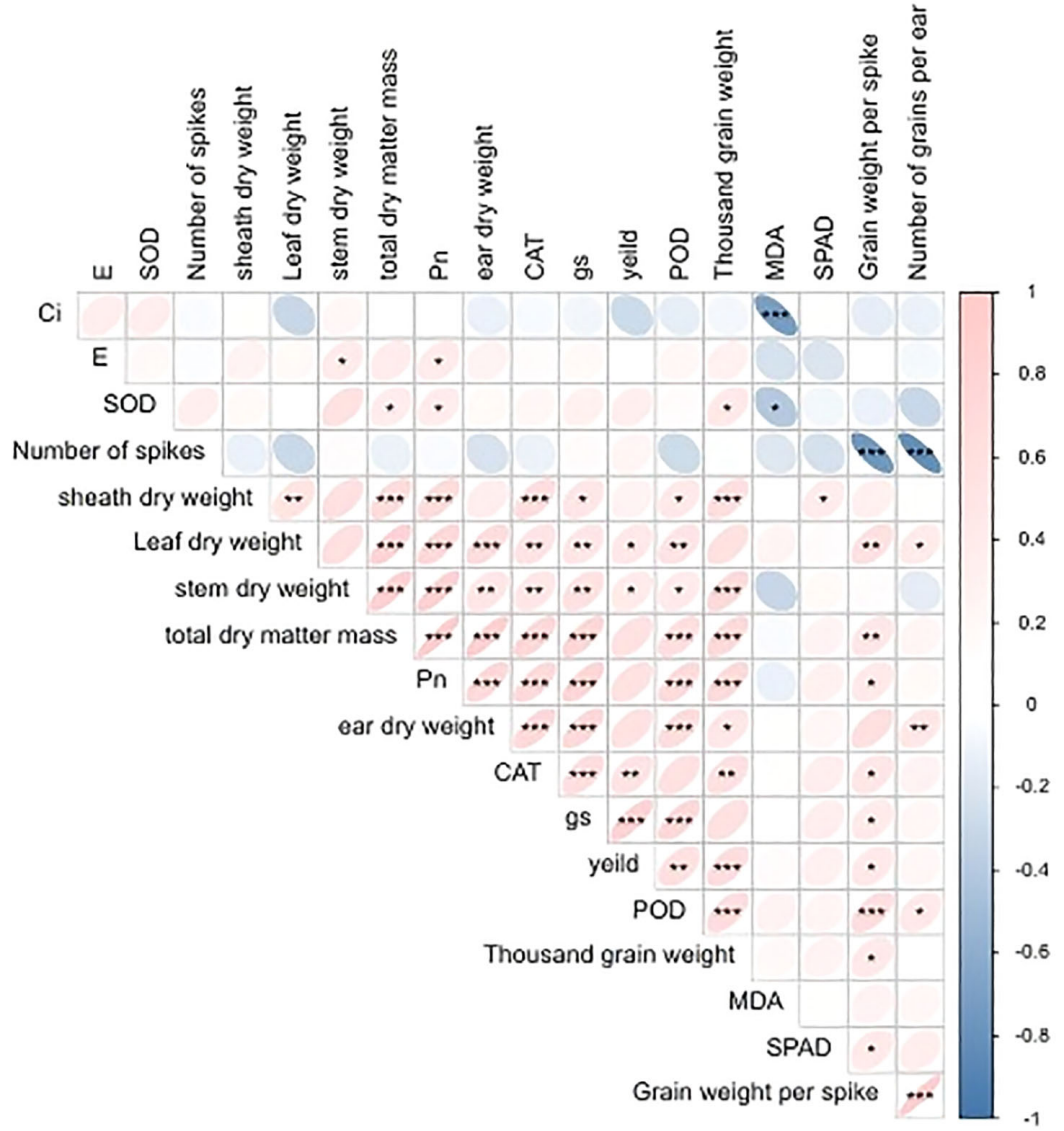
Correlation analysis diagram. * means P≤0.05 level is significant, ** means P≤0.01 level is significant, ***P≤0.001 level is significant.

The results of the correlation analysis, as illustrated in **Figure 4**, revealed a significant negative correlation between Ci, SOD, and MDA. Additionally, a highly significant negative correlation was observed between the number of grains in a spike and both spike weight and thousand grain weight. POD, gs, and yield exhibited a highly significant positive correlation, while leaf and stem dry weights demonstrated a significant positive correlation with yield. The dry weight of a spike, the total dry matter mass, and Pn exhibited a positive correlation with yield, though not to a statistically significant degree. pn and SOD exerted a pronounced influence on the formation of dry matter, and E and SOD exhibited a significant positive correlation with Pn.

## 4 Discussion

### 4.1 Effect of nitrogen and molybdenum fertilizers on yield

Yield is a significant indicator of crop growth and economic value, and photosynthesis in plants exerts a pivotal influence on yield formation (Qing-Hang and Yong-tao, 2019). The results of this study demonstrated that the application of molybdenum and nitrogen fertilizers can enhance the photosynthetic capacity of plants to a certain extent. This enhancement was observed in several key aspects of photosynthesis, including an increase in the net photosynthetic rate and chlorophyll content of the experimental samples. These changes contributed to an overall improvement in the absorption and utilization of photosynthetically active radiation. Secondly, compared with the single application of molybdenum fertilizer, the combined application reduces the intercellular carbon dioxide concentration, reduces the restriction of carbon dioxide during the photosynthesis process, and then improves the photosynthetic efficiency. Additionally, the transpiration rate was found to decrease, which may assist plants in utilizing water more effectively when faced with limited water availability, thereby maintaining elevated photosynthetic activity.These findings align with the impact of *Yunfei* on tobacco photosynthesis when nitrogen and molybdenum fertilizers were applied in conjunction. The enhanced application of molybdenum fertilizer was observed to markedly elevate the photosynthetic rate of leaves across varying nitrogen levels (Zhang et al., 2023).Molybdenum is a vital component in the chlorophyll biosynthesis pathway, the ultrastructure of chloroplasts, and their configuration. It is also an essential trace element for plant photosynthetic processes (Qiang et al., 2015). The primary mechanisms by which this occurs are through participation in nitrogen metabolism, enhancement of chlorophyll synthesis, promotion of the activity of key photosynthetic enzymes, and improvement of plant adaptability to adversity. This, in turn, increases plant photosynthesis and net photosynthesis rates (Saud et al., 2021; Fei et al., 2022; Zhilian et al., 2024). The impact of nitrogen fertilization on photosynthesis is considerable, exerting influence on a number of key processes, including chlorophyll synthesis, the activity of photosynthetic enzymes, and the structure and function of leaves (Dingying et al., 2023; Pengxi et al., 2023).

Furthermore, the thousand kernel weight, yield, and dry matter accumulation of molybdenum and nitrogen-allocated grain Changnong 47 were significantly higher than those of the no molybdenum fertilizer treatment. This finding aligns with the conclusions of Chen Zhiqing in his study on the effect of molybdenum fertilizer on rice yield (Muhammad et al., 2021). A substantial body of research has been conducted on the impact of nitrogen fertilization on plant yield. The application of nitrogen fertilizer has been demonstrated to promote cell division and elongation, as well as the growth of aboveground parts (Lu et al., 2016). This enables the plant to develop a greater number of tissues and organs in a shorter period of time, laying the foundation for enhanced yields at a later stage of the season. It also maintains the equilibrium of nutrients within the plant and stimulates overall growth (Valente et al., 2024; Zhenkun et al., 2024). In the study of the impact of nitrogen application levels on the quality of rice in South China, *Lanlan Zhang* found that the yield of quality rice increased with rising nitrogen application levels (Yanfen et al., 2021). The level of nitrogen applied affects grain yield. Some studies have demonstrated that nitrogen fertilizers are more conducive to grain nitrogen utilization and yield formation (Xuexue et al., 2023; Zu et al., 2024).*Dong Erwei* and colleagues demonstrated that nitrogen application resulted in an increase in the number of spikes harvested, the number of grains per spike, and the plant’s dry matter production capacity in cereal grains. Additionally, they observed an enhanced rate of nitrogen transport from the nutrient organs to the grains and a more efficient allocation of dry matter and nitrogen to the grains, ultimately leading to higher yields (Lanlan et al., 2024). The application of nitrogen fertilizers has been demonstrated to result in enhanced yields of silage corn, increased utilization of nitrogen, improved forage quality, and elevated feed value (Liyuan et al., 2021). The application of molybdenum has been demonstrated to be beneficial for the accumulation of carbohydrates at the tillering and nodulation stages of winter wheat, which in turn promotes an increase in biomass. Furthermore, the transfer of plant growth centers at the spike and grouting stages of the plant has also been shown to be advantageous (Rui et al., 2019).

*Bai Yazheng*’s research on the growth physiology and dry matter accumulation and distribution of cereal grains in molybdenum fertilizer demonstrated a significant positive correlation between the photosynthetic rate and both spike mass and aboveground biomass (Dong et al., 2024). It has been demonstrated that the application of molybdenum fertilizer enhances dry matter accumulation within the plant body by facilitating nitrogen uptake and utilization, thereby improving yield (Bai et al., 2024). The present study did not address the uptake and utilization of nitrogen in cereals under different treatments. This is a topic that will be explored in future research.

### 4.2 Effect of nitrogen and molybdenum fertilizers on antioxidant enzyme activity

Antioxidant enzymes represent a class of enzymes found in plants that are capable of effectively scavenging reactive oxygen species (ROS), including superoxide anion, hydroxyl radicals, and hydrogen peroxide. This process serves to protect plant cells from oxidative damage (Ei et al., 2024). The findings of this study indicate that the application of molybdenum fertilizer in conjunction with nitrogen application resulted in an enhancement of superoxide dismutase (SOD), peroxidase (POD), and catalase (CAT) activities in the cereal grain Changnong 47. This outcome aligns with the existing research on the role of molybdenum in antioxidant enzyme activities in winter wheat (Xuecheng et al., 2006). Nitrogen and molybdenum, in their micronutrient form, are involved in a variety of enzyme activities in plants and affect the synthesis and activity of antioxidant enzymes (Liu et al., 2005; Yang et al., 2023). The addition of nitrogen and molybdenum has been demonstrated to enhance the activity of enzymes involved in antioxidant processes. It has been demonstrated that molybdenum enhances the activities of superoxide dismutase (SOD) and catalase (CAT) in winter wheat subjected to low-temperature stress, thereby augmenting the antioxidant capacity of the plant (Al Issawi et al., 2016). A sufficient supply of nitrogen and molybdenum can enhance the activity of antioxidant enzymes, facilitate the scavenging of reactive oxygen species, mitigate oxidative damage to cells, and bolster plant survival and resilience to oxidative stresses (Kiran et al., 2016).

The increased activity of antioxidant enzymes allows for the more efficient scavenging of excess reactive oxygen species, thereby reducing oxidative damage. Meanwhile, molybdenum affects the activities of key enzymes involved in nitrogen uptake and utilization, such as nitrate reductase (NR) and glutamine synthetase (GS) [25]. These findings provide a foundation for further research in this area.

## Data Availability

The original contributions presented in the study are included in the article/supplementary material. Further inquiries can be directed to the corresponding author.
